# Renal Lipid Metabolism Abnormalities in Obesity and Clear Cell Renal Cell Carcinoma

**DOI:** 10.3390/metabo11090608

**Published:** 2021-09-07

**Authors:** Ion Alexandru Bobulescu, Laurentiu M. Pop, Chinnadurai Mani, Kala Turner, Christian Rivera, Sabiha Khatoon, Subash Kairamkonda, Raquibul Hannan, Komaraiah Palle

**Affiliations:** 1 Department of Cell Biology and Biochemistry, Texas Tech University Health Sciences Center, Lubbock, TX 79430-6540, USA; Chinnadurai.Mani@ttuhsc.edu (C.M.); kala.turner@ttu.edu (K.T.); Christian.Rivera@ttuhsc.edu (C.R.); Sabiha.Khatoon@ttuhsc.edu (S.K.); Subash.Kairamkonda@ttuhsc.edu (S.K.); komaraiah.palle@ttuhsc.edu (K.P.); 2 Department of Internal Medicine, Texas Tech University Health Sciences Center, Lubbock, TX 79430-6540, USA; 3 Department of Radiation Oncology, University of Texas Southwestern Medical Center, Dallas, TX 79430-6540, USA; laurentiu.pop@utsouthwestern.edu (L.M.P.); raquibul.hannan@utsouthwestern.edu (R.H.); 4 Department of Surgery, Texas Tech University Health Sciences Center, Lubbock, TX 79430-6540, USA

**Keywords:** metabolic reprogramming, tumorigenesis, preneoplastic changes, lipotoxicity

## Abstract

Clear cell renal cell carcinoma is the most common and deadly type of cancer affecting the kidney, and is characterized histologically by large intracellular lipid deposits. These deposits are thought to result from lipid metabolic reprogramming occurring in tumor cells, but the exact mechanisms and implications of these metabolic alterations are incompletely understood. Obesity is an independent risk factor for clear cell renal cell carcinoma, and is also associated with lipid accumulation in noncancerous epithelial cells of the proximal tubule, where clear cell renal cell carcinoma originates. This article explores the potential link between obesity-associated renal lipid metabolic disturbances and lipid metabolic reprogramming in clear cell renal cell carcinoma, and discusses potential implications for future research.

## 1. Introduction

Kidney cancer represents approximately 2.2% of all new cancer cases and 1.8% of cancer deaths globally [1]. Approximately 1.7% of all Americans will be diagnosed with kidney cancer during their lifetimes [2]. Renal cell carcinomas (RCCs) are primary adenocarcinomas originating from renal tubular epithelial cells, and are the most common type of kidney cancer (90–95% of all cases). Of these, clear cell renal cell carcinoma (ccRCC), which is thought to arise from proximal tubule epithelial cells [3], is the most common and deadly subtype (75–80% of all cases of RCC) [4,5,6]. Obesity is a major independent risk factor for ccRCC, even after adjusting for potential confounders [7].

The characteristic “clear cell” (or empty cytoplasm) appearance of ccRCC on standard histological examination is due to the intracellular accumulation of large amounts of lipid, and some glycogen, which are washed out during the standard sample preparation procedure [8,9]. These large lipid depots indicate that lipid metabolic reprogramming is a central feature of ccRCC, but the roles played by lipid accumulation in the biology and pathogenesis of ccRCC remain incompletely understood.

Multiple studies in humans and animal models have shown that renal lipid accumulation also occurs in obesity, in the absence of ccRCC, and can lead to toxic effects termed lipotoxicity [10,11,12]. Lipid accumulation in non-adipose tissues has been linked with tumorigenesis in other organs, such as the liver [13] and pancreas [14], but whether lipid accumulation and lipotoxicity in non-cancerous renal cells may precede neoplastic changes and may play a role in the pathogenesis of ccRCC has not been examined.

In this article, we review the pathophysiologic mechanisms and implications of lipid accumulation in ccRCC and in non-cancerous proximal tubule cells, and propose a new research direction examining the link between obesity-related renal lipid accumulation and ccRCC pathogenesis.

## 2. Lipid Uptake and Metabolism in Normal Proximal Tubule Cells

Fatty acids are a major source of energy for normal renal proximal tubule cells, with mitochondrial fatty acid oxidation supplying more than half of the vast amount of ATP required to fuel proximal tubular renal sodium reabsorption [15,16].

Although the exact mechanisms of fatty acid uptake in the human proximal tubule remain a subject of debate, it appears that cluster of differentiation 36 (CD36), also known as fatty acid translocase, is a key player in the import of long-chain fatty acids from the circulation [17,18]. The expression of CD36 is markedly increased in hyperlipidemia [19], which may explain at least in part the fact that renal uptake of fatty acids is linearly dependent on plasma fatty acid concentrations [16]. In addition, proximal tubule cells take up fatty acids carried on albumin from the luminal side by receptor-mediated endocytosis [20,21], as well as via the apical membrane fatty acid transporter-2 (FATP2) protein [22]. While apical uptake of fatty acids from the primary urine likely represents less than 5% of the total fatty acid uptake in proximal tubule cells under normal circumstances, apical uptake can increase markedly in pathologic conditions, such as hyperlipidemia and proteinuria [23]. Taken together, these characteristics make the proximal tubule cell particularly susceptible to imbalances between lipid uptake and utilization, which may result in lipid accumulation and lipotoxicity [23].

Whether de novo lipid synthesis occurs in proximal tubule cells under normal circumstances is less clear. Current evidence suggests that de novo lipid synthesis in these cells is unlikely to be physiologically significant, since proximal tubule cells can flexibly utilize both fatty acids and glucose to generate ATP; they do not appear to release fatty acids into the circulation [15]; and they have very low expression of fatty acid synthase [24,25].

## 3. Lipid Metabolic Disturbances in Non-Cancerous Proximal Tubule Cells

The hypothesis that dyslipidemia may contribute to kidney dysfunction (termed “lipid nephrotoxicity”) was first proposed by Moorhead and colleagues almost four decades ago [26]. Dyslipidemia may cause an imbalance between lipid provision and lipid utilization in renal cells, with potential toxic effects [27], and may also affect the kidney indirectly through mechanisms, such as systemic inflammation, vascular injury, and endocrine abnormalities [28,29].

An imbalance between lipid availability and utilization in renal tubular cells most commonly manifests as renal lipid accumulation, which has been described in multiple animal models of obesity, diabetes, and renal injury [23], as well as in humans with obesity and diabetes mellitus [10,11,12]. Uptake of fatty acids in proximal tubule cells is increased with increases in plasma concentration of fatty acids, is associated with significantly upregulated expression of the fatty acid transporter CD36, and may at least in part underpin renal lipid accumulation is disease states, such as obesity and diabetes [16,19].

Renal lipid accumulation in certain animal models was also linked with changes in the activity of sterol regulatory element-binding proteins (SREBPs), the “master regulators” of lipid biosynthesis, suggesting a possible role of de novo lipid synthesis in renal lipotoxicity [30,31]. Pharmacologic prevention of lipid accumulation in the proximal tubules of obese rodents with type 2 diabetes, using peroxisome proliferator-activated receptor-gamma agonists, prevented alterations in renal tubular function [32]. Conversely, lipid accumulation associated with decreased utilization of fatty acids has been described in humans and animals with tubulointerstitial fibrosis, and restoring fatty acid oxidation by genetic or pharmacologic methods protected mice from fibrosis [33].

The mechanisms linking lipid accumulation to cellular injury (lipotoxicity) and dysfunction in the kidney and other organs have been reviewed extensively [27,34,35,36,37,38]. Lipid accumulation per se is not thought to be deleterious to the cell (except in extreme cases) but serves as an indicator of disturbed lipid metabolism and as a dynamic reservoir of excess fatty acids. In turn, excess intracellular fatty acids may have multiple toxic effects, including mitochondrial damage, oxidative stress, endoplasmic reticulum stress, and proinflammatory signaling. These effects vary depending on cell type, beta-oxidative and lipogenic capacities, and the relative intracellular abundance of different species of fatty acids.

Our knowledge of these processes comes primarily from in vitro and in vivo laboratory studies and from small translational studies including human subjects or tissue specimens, with only limited data from human trials. Randomized controlled trials with prespecified primary renal outcomes failed to demonstrate a beneficial effect of HMG-CoA reductase inhibitors (statins) on kidney function [39,40], but those trials had limited statistical power and were restricted to patients already diagnosed with chronic kidney disease (CKD). A recent post-hoc pooled analysis of 6 large randomized trials encompassing over 30,000 participants with or without CKD reported significant and dose-dependent renoprotection with statin treatment [41], and a meta-analysis of 43 randomized trials encompassing over 110,000 participants showed that statin treatment slowed the progression of kidney function decline [42]. Although statins have been shown to reduce proximal tubule lipid accumulation in rodents with diet-induced obesity [43], the extent to which statins may affect kidney function by reducing renal lipotoxicity, versus through other indirect or pleiotropic mechanisms, remains unknown.

## 4. Lipid Metabolic Reprogramming in ccRCC

Cancer cell metabolic reprogramming is now recognized as one of the essential hallmarks of cancer [44,45]. The first description of metabolic reprogramming was published almost a century ago by Warburg and colleagues, who showed a preference for glycolysis in tumor cells even in the presence of oxygen (aerobic glycolysis) [46,47]. Almost seven decades ago, Mendes and colleagues demonstrated that lipid metabolism is also reprogrammed in cancer, with neoplastic tissues gaining the ability to synthetize lipids de novo [48]. Various alterations of lipid metabolism have since been described in most cancers, even in the absence of overt lipid accumulation [49,50,51]. Altered lipid metabolism is essential for tumor progression, partly due to the increased demand for phospholipids to build new cell membranes, but also because of metabolic changes favoring other aspects of tumor growth and pro-oncogenic signaling within cancer cells and in the tumor microenvironment [50,51,52,53].

With only a few exceptions, the metabolic needs of normal non-cancerous cells are satisfied by the uptake of lipids from the circulation, with circulating lipids originating from diet, hepatic synthesis, or adipose tissue catabolism. While lipid uptake remains active and may even be upregulated in some cancers [54,55,56], most cancer cells also gain the ability to synthetize lipids, including fatty acids and cholesterol, from non-lipid substrates [53,57]. In addition, cancer cells may either activate or repress fatty acid beta-oxidation, may alter the fatty acid composition of various cellular lipids, and may also store excess fatty acids as triglycerides in intracellular lipid droplets [51,57].

Alterations of lipid metabolism described in ccRCC include increased uptake of extracellular lipids [58], tumor grade-dependent increased de novo synthesis of fatty acids [59] and decrease in fatty acid beta-oxidation [60,61,62], and the pathognomonic accumulation of large intracellular lipid deposits [8,9]. Untargeted lipidomic analyses of ccRCC compared with normal kidney tissue revealed a characteristic “ccRCC lipidomic signature”, including increased levels of cholesteryl esters and triacylglycerols, as well as lower levels of phospholipids (other than phosphatidylcholines) and polyunsaturated fatty acids [63,64]. Furthermore, integrated lipidomic and transcriptomic analyses showed that alterations in cellular lipid composition in ccRCC were accompanied by multiple consistent changes in gene expression, including increased CD36 (fatty acid uptake), stearoyl-CoA desaturase and fatty acid elongases 2 and 5 (fatty acid synthesis), reduced carnitine palmitoyltransferase 1A (CPT1A, rate-limiting enzyme for the transport of fatty acids into mitochondria for beta-oxidation), as well as perilipin 2 (triglyceride storage in lipid droplets) [64]. These alterations likely play important roles in ccRCC biology, and are logical therapeutic targets for ongoing and future investigation.

In addition to disturbances in the metabolism of fatty acids, recent findings suggest that ccRCC may be dependent on the uptake of exogenous cholesterol. High-density lipoprotein (HDL) cholesterol uptake may contribute to both lipid accumulation and tumor growth and survival in ccRCC [58,65,66], and targeting cholesterol uptake via the scavenger receptor B1 (SCARB1) may be a novel therapeutic target in ccRCC [66], although this requires further investigation. Additionally, data from large retrospective studies suggest that statin treatment may be associated with decreased risk of ccRCC [67,68]. While these findings are not definitive [69], they are compatible with a potential role of low-density lipoprotein (LDL) cholesterol in ccRCC pathogenesis. Statins have also been postulated to suppress ccRCC by targeting the mevalonate pathway in tumor cells, which disrupts the prenylation of small GTPases and impairs cancer cell signaling [70].

Some lipid metabolic changes have been directly linked to the dysfunction of genes that are characteristically altered in ccRCC. For instance, loss of functional von Hippel-Lindau (VHL) tumor suppressor gene is the most common genetic alteration occurring in ccRCC (up to 90% of cases) [71,72]. While loss of VHL alone is insufficient to cause ccRCC in the absence of a “second-hit” event [73,74], decreased VHL activity leads to the stabilization of hypoxia-inducible factors (HIF1 and HIF2) and their constitutive activation [75]. Loss of the second most mutated gene in ccRCC, polybromo 1 (PBRM1), has been shown to contribute to the carcinogenesis process by amplifying the HIF response to VHL loss [76,77].

Hypoxia-inducible factors are essential for cellular adaptation to hypoxia and are master regulators of multiple metabolic pathways in both normal and cancerous cells, including glycolysis, glycogenesis, the tricarboxylic acid cycle, and lipid metabolism [78,79]. HIF activation in ccRCC represses CPT1A-mediated fatty acid transport into mitochondria, thus decreasing fatty acid beta-oxidation [61]. HIF activation may also inhibit medium- and long-chain acyl-CoA dehydrogenases (MCADs and LCADs), as described in hepatocellular carcinoma [80]; may drive increased fatty acid synthesis via activation of Akt and sterol regulatory element binding protein-1 (SREBP-1), as described in breast cancer cells [81]; and may thus drive intracellular lipid accumulation via multiple pathways [78]. Of note, HIF activation in ccRCC may occur even in the absence of VHL loss, possibly driven by hypoxia alone [82], and thus the extent to which VHL inactivation is responsible for de novo fatty acid synthesis, decreased beta-oxidation, and lipid accumulation is unclear.

Another gene frequently mutated in ccRCC, the SET domain containing 2 histone lysine methyltransferase (SETD2), has been linked with alterations in lipid metabolism in the liver, in addition to its roles in DNA repair and genomic stability [83]. When Setd2-null mice were fed a high-fat diet, this resulted in increased hepatic lipid accumulation and hepatocellular carcinoma [83]. However, whether SETD2 might affect renal lipid metabolism and ccRCC tumorigenesis in a similar manner remains unclear.

The links between ccRCC genetic alterations and increased lipid uptake are also unclear. In addition to CD36, there is evidence that the fatty acid transporter FATP4 (normally expressed at very low levels in the kidney) is a biomarker of poor prognosis in patients with ccRCC [84,85]. In turn, FATP4 is a transcriptional target of HIF-1 [86].

Of note, while the role of lipid accumulation in ccRCC tumorigenesis and progression remains incompletely understood, accumulation of triglycerides has been shown to make ccRCC cells more susceptible to ferroptosis [87,88], a form of regulated cell death that is mediated by iron-dependent accumulation of lipid hydroperoxides [89]. Cancer cells upregulate the glutathione/glutathione peroxidase (GPX) redox system to prevent ferroptosis, and interference with this system may be a therapeutic target in ccRCC [87,88].

## 5. Exploring the Link between Obesity and Renal Cell Carcinoma

Obesity, smoking, and hypertension are the most important modifiable risk factors for RCC, and appear to increase cancer risk independently of each other [7,90,91,92]. In both men and women, obesity was associated with increased risk of RCC even after adjusting for age, race/ethnicity, blood pressure, and smoking status [92,93]. Based on obesity prevalence data for the years 1999–2000 in the National Health and Nutrition Examination Survey (NHANES), obesity was estimated to contribute to over 40% of ccRCC cases in the United States [91]. Since any such estimate is highly dependent on the prevalence of obesity in a given population, the proportion of ccRCC attributable to obesity is likely to increase as the prevalence of obesity increases in the U.S. and worldwide [94]. The prevalence of obesity has nearly tripled globally since 1975 [95], and now exceeds 40% of the adult population in the United States [96].

Of note, while a large body of evidence strongly supports the notion that obesity increases the risk of incident ccRCC in the general population, elevated BMI has also been associated with better prognosis among those patients who are already diagnosed with ccRCC [97,98,99]. This “obesity paradox” is not unique to kidney cancer, having been described in other neoplasms, such as lung cancer and melanoma [100,101,102], as well as in non-neoplastic diseases, such as chronic heart failure and coronary artery disease [103]. Obesity appears to be associated with better outcomes in those patients with RCC who undergo surgical or pharmacologic treatment, with less evidence available for such an effect in patients with untreated RCC [104,105]. Multiple mechanisms have been proposed to explain the obesity paradox in RCC, including differences in the tumor microenvironment and in the peritumoral adipose tissue, differences in local inflammatory responses, and the action of adipokines [106,107,108]. There is also ongoing controversy in the field, with some authors contending that the obesity paradox is primarily related to methodological pitfalls in existing clinical studies, such as collider stratification bias, detection bias, or reverse causation [109]. Although a detailed discussion of the obesity paradox is beyond the scope of this article, it is important to note that any survival benefit associated with elevated BMI in patients already diagnosed with RCC does not negate or contradict the vast body of evidence indicating that obesity is a major independent risk factor for RCC in the general population.

Obesity also increases the risk of other cancers, with more than a dozen cancer types considered obesity-related by the World Health Organization’s International Agency for Research on Cancer (IARC) [110]. A number of mechanisms by which obesity may increase cancer risk have been proposed, including chronic inflammation, oxidative stress, endocrine disturbances (insulin, insulin-like growth factor 1, gonadocorticoids), changes in adipokine levels (leptin, adiponectin), and changes in the gut microbiota [111,112,113]. However, little is known about the mechanisms linking obesity to the pathogenesis of ccRCC.

Inflammatory markers that are increased in obesity, such as tumor necrosis factor-α (TNF-α) and interleukin-6 (IL-6), were associated with increased risk of incident ccRCC, but whether these inflammatory pathways play a causal role in the pathogenesis of ccRCC has not been definitively established [114,115]. The interplay between inflammation and ccRCC is likely to be highly complex, and increased peritumoral adipose tissue inflammation (as reflected by transcriptomic data) was also proposed to contribute to better prognosis in obese patients undergoing either surgical or pharmacologic treatment for ccRCC (the “obesity paradox” discussed earlier in this article) [106].

Associations between the risk of RCC and circulating adipokines that are altered in obesity were described in some studies but not in others. For instance, a case-control study of 546 Finnish male smokers found that low levels of serum adiponectin (common in obesity) were associated with increased risk of RCC even after adjusting for potential confounders including body mass index (BMI) [116], and another report from the same group found associations between both leptin and adiponectin concentrations and the risk of RCC in a case-control study enrolling more than 1600 African American and Caucasian participants from two urban areas in the United States [117]. However, a more recent case-control study enrolling 504 participants did not find significant associations between the risk of RCC and serum adiponectin levels, [118], and a large Mendelian randomization study including 10,784 RCC cases and 20,406 controls found no association between the risk of RCC and leptin or adiponectin [119].

## 6. Alterations in Renal Lipid Metabolism: A Potential Link between Obesity and ccRCC

Another possible mechanism linking obesity to ccRCC risk may be inferred from the fact that non-cancerous proximal tubule cells in obesity suffer alterations in lipid metabolism that are strikingly similar to ccRCC, including induction of de novo lipid synthesis and lipid accumulation [10,11,12,30]. Pathologic lipid accumulation has been linked with tumorigenesis in other organs, including the liver [13] and pancreas [14]. In the liver, alterations in lipid metabolism leading to lipid accumulation and lipotoxicity have been proposed to play a role in the transition from alcohol-related liver disease, non-alcoholic fatty liver disease (NAFLD), and non-alcoholic steatohepatitis (NASH) to hepatocellular carcinoma [120,121,122].

**Figure 1:**
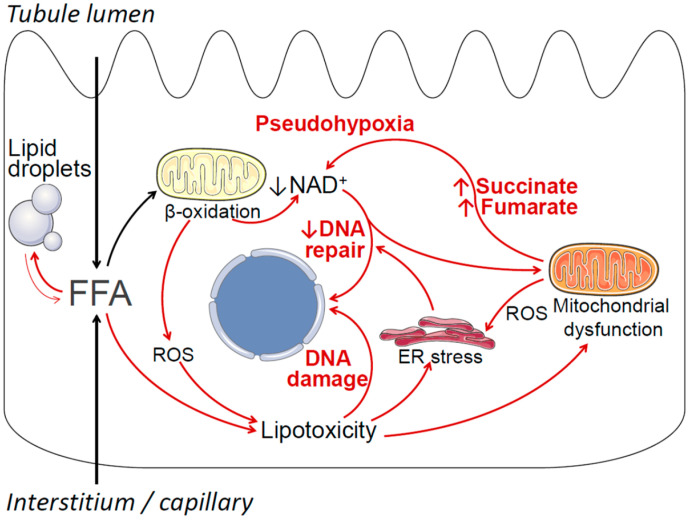
Summary of potential mechanisms by which obesity-related lipid metabolic disturbances in renal proximal tubule cells may contribute to cancerous cell transformation and ccRCC. Free (non-esterified) fatty acids (FFAs) enter proximal tubule cells via the basolateral side (from the circulation) and the apical side (reabsorption of albumin-bound fatty acids). Under normal conditions most FFAs are transported into the mitochondria, where they undergo beta-oxidation, thus fulfilling the proximal tubule’s large ATP requirements. Excess FFA resulting in increased beta-oxidation may deplete cellular nicotinamide adenine dinucleotide (NAD^+^), resulting in a pseudohypoxic state, which in turn may lead to VHL-independent HIF activation. Declining NAD^+^ levels may also negatively affect NAD^+^-dependent cellular DNA repair and DNA damage response mechanisms, including PARP enzymes and sirtuins. Increased beta-oxidation also results in increased production of reactive oxygen species (ROS). When the availability of FFAs exceeds the cells’ beta-oxidative capacity, FFAs may be stored in lipid droplets, and may also enter alternative metabolic pathways, resulting in the formation of toxic compounds, such as ceramides and diacylglycerol. These compounds, together with increased ROS, exert a variety of toxic effects collectively known as lipotoxicity, resulting in DNA damage, endoplasmic reticulum (ER) stress, and mitochondrial dysfunction. In turn, ER stress may further impair the cell’s DNA repair and DNA damage response mechanisms, and mitochondrial dysfunction may result in increased generation of pro-oncogenic metabolites, such as succinate and fumarate, that contribute to pseudohypoxia.

Potential mechanisms by which obesity-associated lipid accumulation and lipotoxicity may contribute to cancerous cell transformation and ccRCC include oxidative stress, endoplasmic reticulum (ER) stress, mitochondrial dysfunction, increased generation of pro-oncogenic metabolites, DNA damage, and negative modulation of DNA repair pathways [123,124,125,126,127,128]. These mechanisms are summarized in Figure 1. Briefly, obesity is associated with increased uptake of free fatty acids (FFAs) into proximal tubule cells from the circulation, as well as increased uptake of FFAs carried on albumin from the tubule lumen [23]. This oversupply of FFAs leads to increased mitochondrial beta-oxidation of FFAs for ATP production, which in turn may result in declining cellular nicotinamide adenine dinucleotide (NAD^+^) levels, as well as increased production of reactive oxygen species (ROS) [129]. Declining NAD^+^ levels induce a state of cellular pseudohypoxia [130], which may lead to HIF activation independent of VHL [131]. Declining NAD^+^ may also affect the function of NAD^+^-dependent enzymes that are essential for DNA repair, such as sirtuins and poly (ADP-ribose) polymerases (PARP) [132]. When FFA oversupply exceeds the cell’s beta-oxidative capacity, excess FFA is stored in lipid droplets (i.e., lipid accumulation), and may also enter alternative metabolic pathways, resulting in the formation of lipotoxic metabolites, such as ceramides and diacylglycerol [37,133]. Lipotoxicity, oxidative stress, and lipid peroxidation may lead to DNA damage, endoplasmic reticulum (ER) stress, and mitochondrial dysfunction [134,135,136,137]. In turn, ER stress may further impair cellular DNA repair mechanisms [138], and mitochondrial dysfunction may result in the overproduction of pro-oncogenic metabolites, such as succinate and fumarate, that further contribute to cellular pseudohypoxia [128]. Further detailed studies are required to investigate the potential contribution of these mechanisms, individually and in combination, to ccRCC tumorigenesis. Importantly, the overarching hypothesis that obesity-associated lipid accumulation may play a causal role in the pathogenesis of ccRCC does not require all elements of Figure 1 to be true, and the model will likely be revised as new data emerge.

## 7. Conclusions

Lipid metabolic reprogramming and lipid accumulation are hallmarks of ccRCC, and show similarities with obesity-associated lipid disturbances in non-cancerous renal proximal tubule cells. Additional studies are required to better understand these similarities, to characterize preneoplastic cellular changes in obesity-associated renal lipid accumulation, and to investigate the potential mechanisms linking lipid disturbances with tumorigenesis in ccRCC (Figure 1). In addition to defining the pre-cancerous role of renal lipid disturbances in patients with obesity, future research efforts may identify novel therapeutic approaches targeting renal lipid metabolism to prevent and treat ccRCC, as well as potential lipid-related biomarkers that could guide precision medicine interventions.
